# Beef Tea

**Published:** 1903-01-24

**Authors:** 


### EDITOR'S LETTER-BOX.

**[Our Correspondents are reminded that prolixity is a great bar to publication, and that brevity of style and conciseness of statement greatly facilitate early insertion.]**

BEEF TEA.

BOVRIL, LIMITED, write:—In continuing the discussion originated by us, advocating for economic and scientific reasons the use of concentrated meat preparations such as ours instead of beef tea, your correspondent "S. H.", while admitting the nutritive element in Bovril, and thus agreeing on this point with the matrons whose experience has led them to corroborate our statements, falls, inadvertently no doubt, into an error which we desire to correct. "S. H." labours under the impression that we suggest the use of Bovril for sick dietary to the exclusion of all other proteid-containing food. This, of course, is quite an erroneous assumption. For the reasons advanced by Dr. Hutchison it would be undesirable, as your foot-note very properly shows, to use even the elaborately prepared beef tea, referred to by Dr. Hutchison, as the sole source of proteid.

Apart from our own personal interest in the matter, we really think that the present time, when the question of economy is in the air, seems opportune for inquiring if the expenditure on beef tea, which forms so large an item in hospital outlay, could not be reduced without detriment to the welfare of the patients by using concentrated preparations vastly superior, cheaper, and involving practically no expense or trouble in the making. We could mention numerous institutions where reforms have already taken place in this direction with the approval of the medical staffs, and with great benefit to their funds, and "S H." or any other bona fide matron or hospital official communicating with us on the subject can be referred to them.

